# The Glandular Fever of Childhood

**Published:** 1897-08

**Authors:** 


### THE GLANDULAR FEVER OF CHILDHOOD.

Dawson Williams reports two cases of the above-named affection. E. Pfeiffer, in 1889, described a condition observed in childhood characterized by acute enlargement of the glands which he contended was an acute specific fever, hitherto unrecognized, and to which he gave the name "glandular fever" (Drusenfieber). The general symptoms described by Pfeiffer, and confirmed by later observers, are as follows: The patient, a child under 14 years, becomes suddenly ill; the temperature rises to 101° to 103° F.; there are anorexia, nausea, sometimes vomiting, coated tongue, constipation, and occasionally ill-defined abdominal pains. The most prominent and characteristic symptoms, however, are stiffness of the neck, tenderness in the anterior triangle, and pain on movement of the head, and on deglutition. The pharyngeal mucous membrane may be slightly reddened, but at no time during the course of the illness is there a definite tonsillitis or pharyngitis. On the second or third day a swelling is noticed in the neck, due to three or four enlarged lymphatic glands, which can be felt beneath the sternocleido-mastoid muscle and the anterior border. The temperature usually becomes higher at this time, and generally reaches 104° F. The tender glands remain swollen for from two to five days, and then begin to diminish. The glands on the left side are nearly always first involved, and before they begin to subside those on the right side commence to enlarge, and later reach a size equal to those first involved. Abdominal tenderness may be marked, and in the majority of the cases the mesenteric glands may be felt to be enlarged. Liver and spleen are enlarged in more than one-half the cases. The axillary and inguinal glands are swollen in some instances. The disease is usually mild, and seldom, if ever, is the direct cause of death. The glandular enlargement usually disappears within ten to fourteen days. Quite marked anemia and weakness may persist for some time after the acute symptoms have disappeared.

The two cases reported by Williams were children in the same family, and presented the characteristics above described, the glandular enlargement commencing on the left side, and later appearing on the right, without any associated throat symptoms. A third child in this family had just previously suffered a similar attack, suggesting an infectious character. Pfeiffer observed that the disease occurred in very limited epidemics, generally affecting a single family, but attacking most of the members who had not passed childhood. Those who have described the disease since Pfeiffer have laid great stress on the absence of local throat symptoms to account for the glandular enlargement. Dr. Park West reported ninety-six cases that occurred in eastern Ohio from 1893 to 1896. They presented the general features already related. Only one case was fatal, a delicate child convalescing from scarlet fever. Hœrschelmann thinks the incubation period is usually from eight to ten days. West observed that most of his cases showed signs seven days after exposure. Suppuration is extremely rare, although Neumann and Comby have reported cases in which it occurred. Hesse and Starck observed severe nephritis as a complication in several members of same family.

As to the pathology of the disease little is known, and opinions vary considerably. Comby is of the opinion that it is due to an attenuated streptococcus infection, the surface of the tonsils constituting the point of entery. He thus favors the view that the disease is an acute specific infection, with which Ashby and Wright agree. The constant association of constipation led Starck to advance the theory that the infection might be of intestinal origin, due to absorption of toxins from the retained feces. Koplik suggests that the earlier involvement of the glands on the left side might be due to the passage of the infective agent from the thoracic duct to the glands on the same side. Williams thinks that the infective agent, whatever it may be, most probably enters by the pharynx or tonsils, without producing a local lesion at the point of entrance.—*Lancet.*

